# The Possibility of Decreasing 50-Hz Electric Field Exposure near 400-kV Power Lines with Arc Flash Personal Protective Equipment

**DOI:** 10.3390/ijerph13100942

**Published:** 2016-09-23

**Authors:** Leena Korpinen, Herkko Pirkkalainen, Timo Heiskanen, Rauno Pääkkönen

**Affiliations:** 1 The Clinical Physiology and Neurophysiology Unit, The North Karelia Central Hospital and Honkalampi Centre, Tikkamäentie 16, FIN-80210 Joensuu, Finland; 2 Fingrid Oyj, Wredenkatu 2, 78250 Varkaus, Finland; herkko.pirkkalainen@gmail.com (H.P.); timo.heiskanen@fingrid.fi (T.H.); 3 Tampere University of Technology, Korkeakoulunkatu 10, 33720 Tampere, Finland; rauno.paakkonen@gmail.com

**Keywords:** power lines, electric field, exposure

## Abstract

Various guidelines for the protection of human beings against possible adverse effects resulting from exposure to electromagnetic fields (EMFs) have been published with a view towards continual improvement; therefore, decreasing exposure is an important research area. The aim of this study was to investigate the possibility of decreasing electric field exposure with arc flash rated personal protective equipment (PPE), which in this case was a set of coveralls, and to compare the measurement results to calculations using the helmet-mask measuring system. We collected the data under a 400-kV power line. The test person stood on isolated aluminum paper, and the current between the ground and the aluminum paper was measured. When the test subject wore the arc flash PPE, the current to the ground was only 9.5% of the current measured when wearing normal clothes, which represents a clear decrease in exposure.

## 1. Introduction

When electricians work under high voltage power lines or in electrical substations, they are exposed to an alternating, very low frequency electric field. The alternating electric field induces a surface electric charge and a current in the worker’s body, which leads to a current flowing between the worker and the ground through any contact point, e.g., the feet or hands that touch railings. Typically, the workers are not grounded with a conductor and do not wear equipment that would specifically protect them from exposure to the electric field. Occupational and public electric field exposures have been studied earlier in Finland [1,2,3,4,5,6,7,8]. For example, cardiac pacemakers and implantable cardioverter defibrillators in the electric fields of 400-kV power lines have been tested [1,2]. The risk of the disturbances on pacemakers is not deemed to be high because only one type out of several tested pacemakers showed a major disturbance in a unipolar electrode configuration. In addition, during the experiments, anomalous behavior was observed in only one implantable cardioverter defibrillator [2].

Various legislation and guidelines of occupational electromagnetic field exposure have been published: (1) directive 2013/35/EU on the minimum health and safety requirements regarding the exposure of workers to the risks arising from physical agents (electromagnetic fields) [9]; (2) the guidelines of the International Commission on Non-Ionizing Radiation Protection (ICNIRP) [10,11]; and (3) the IEEE Standard for Low-Frequency Narrowband Power Line Communications for Smart Grid Applications [12].

Directive 2013/35/EU includes minimum requirements for the protection of workers from risks to their health and safety arising from exposure to electromagnetic fields at their work sites. The action levels (ALs, workers) of the directive regarding electric fields (50 Hz) are as follows: low AL 10 kV/m (rms) and high AL 20 kV/m (rms) [9].

Indirect effects of electromagnetic fields may result from physical contact between a person and an object of a dissimilar electric potential. This causes a flow of electric charge (contact current). In the frequency range up to approximately 100 kHz, the flow of electric current from an object in the field to the body of an individual may result in the stimulation of muscles, peripheral nerves, or both. With increasing levels of current, this may be manifested as perception, pain from electric shock, burn, or both, inability to release the object, difficulty in breathing, and, at very high currents, cardiac ventricular fibrillation [13]. Threshold values for these effects are lowest at frequencies between 10 and 100 Hz. Spark discharges can occur when an individual comes into very close proximity with an object of a different electric potential without actually touching it [3,10]. Indirect effects for the threshold current (mA) at a 50-Hz frequency are touch perception (0.2–0.4 mA), pain on finger contact (0.9–1.8 mA), painful shock (8–16 mA), and severe shock (12–23 mA) [11]. The reference level for time varying contact currents from conductive objects for occupational exposure of contact current is 1.0 mA [11,12]. Engineering controls and wearing personal protective clothing can prevent these problems from occurring.

A study published in 1996 examined statistical data collected from French electric utility workers. It showed that being exposed to a very low frequency electric field may increase the risk of brain tumors, and an association with colon cancer was also found. Links to other kinds of cancers were not found [14]. A meta-analysis published in 2001 also concluded that the risk of brain tumors is slightly increased in workers continually exposed to very low frequency electric fields [15].

In a previous study conducted at Tampere University of Technology, the maximum total contact currents of workers were measured using a helmet–mask measuring system at 400-kV substations and under 400-kV power lines [4]. The helmet-mask measuring system consists of a special helmet and two multimeters carried in a waist bag by the test subject. The helmet is covered in copper foil and has a visor made out of a steel net surrounding the head of the test subject. The system is grounded by a conductor wire. One of the multimeters is connected to the helmet and measures the current between the helmet and the ground. The other multimeter is connected to the test subject’s arm with an electrode and measures the current between the entire test subject and the ground. Part of this study, the possibility of decreasing the 50-Hz electric field exposure with different coveralls and under 400-kV power lines, was explored in 2015 [5]. Tampere University of Technology tested three different coveralls under a 400-kV power line, with a distance of 5 m between the probe and the closest overhead conductor. The test subject put on different coveralls, and she stood on aluminum paper, which was isolated from the ground with a plastic bag. The test subject did not wear shoes or socks, and the current between the ground and the aluminum paper was measured. The results were as follows: (1) with normal clothes, it was 41.4 μA; (2) with conductive textile coveralls (special clothing that decreases electric field exposure), 3.2 μA; (3) with coveralls (worn by electric company workers), 44.2 μA; and (4) with coveralls (for winter, worn by workers), 46.0 μA. The current from the test subject to the ground (with conductive textile coveralls) was only 7.7% of the current measured when wearing normal clothes. The electric field was 3.8 kV/m (a height of 1.7 m).

In another study at Tampere University of Technology on the possibility of decreasing the 50-Hz electric field exposure with different jackets was studied under a 400-kV power line using a wooden frame from which the jackets could be suspended [6]. The electric field was 3.2 kV/m (a height of 1.4 m), near the frame. Tampere University of Technology tested a summer jacket, a winter jacket, and a vest. The sensor of a three-axis commercial EFA-300 meter was inside the jackets, which measured the fields. The measured electric fields in the following tests were as follows: (1) the summer jacket was 3.2 kV/m; (2) the winter jacket was 1.3–1.4 kV/m; and (3) the vest was 3.4 kV/m. When the winter jacket was grounded, the electric field was 2.5 kV/m. The winter jacket marginally decreased the electric field exposure inside the jacket [6].

The aim of this study was to investigate the possibility of decreasing the 50-Hz electric field exposure with arc flash personal protective equipment (PPE) under 400-kV power lines and to compare the measurement results to those of the helmet-mask measuring system. Furthermore, the aim was to compare the new results to earlier measurement results of coveralls under 400-kV power lines.

## 2. Materials and Methods 

**Figure 1:**
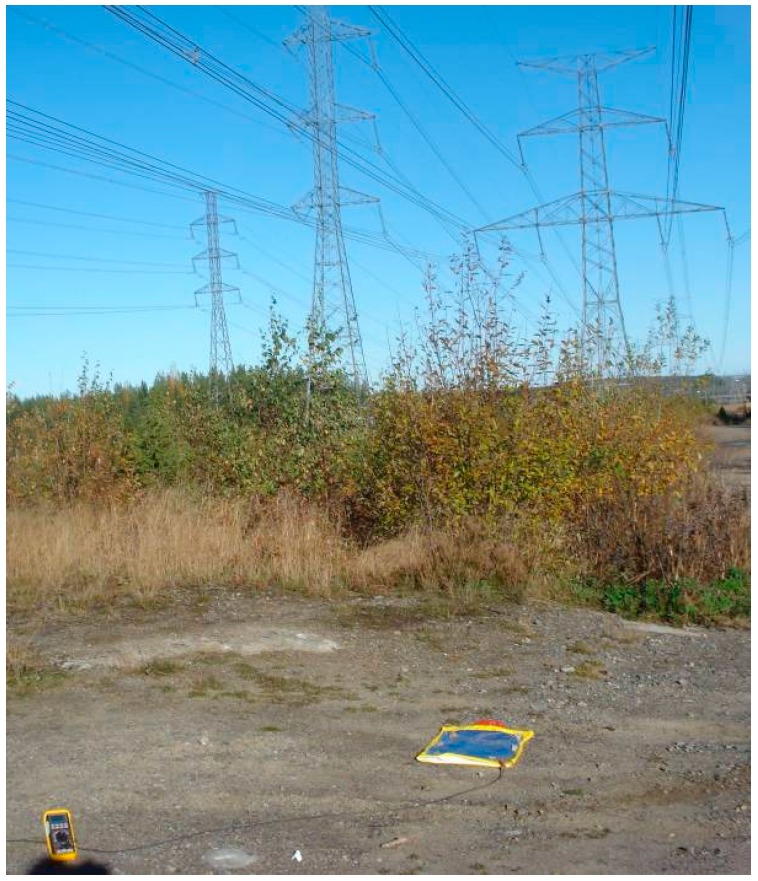
Experiment location.

Figure 1 shows the measurement location. The experiments were performed at the same place as in the earlier study for the sake of consistency [5]. The location is conveniently situated and easily accessible, which allows for the scheduling of multiple measurement days in case of bad weather.

**Figure 2:**
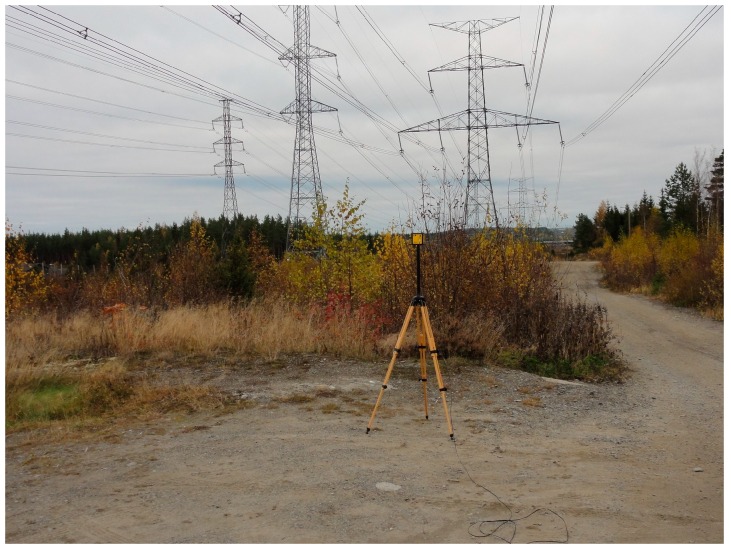
Electric field measurement at experiment location.

First, we measured the electric field at the experiment site (Figure 2). We performed the electric field measurements with a commercial EFA-300 meter, using the three-axis E-Field Probe 2245-302 attachment (accuracy: 3%, rms) calibrated by the manufacturer (Narda Safety Test Solutions GmbH, Pfullingen, Germany). The frequency range was 5–30 kHz, and the measurement height was 1.7 m. The distance between the probe and the closest overhead conductor was approximately 5 m. The average current load and voltage at the time of the measurements were 495.1 MVA and 412.5 kV for the first day and 570.2 MVA and 411.7 kV for the second day.

**Figure 3:**
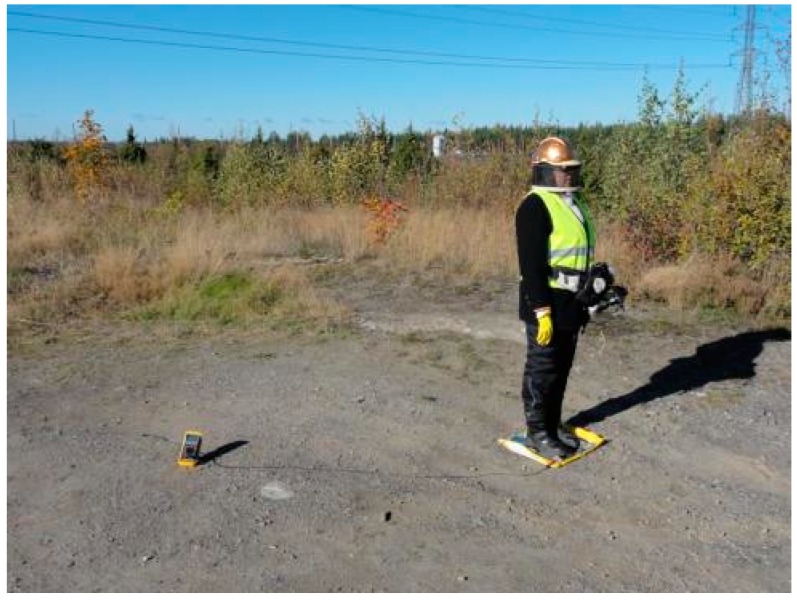
Test subject using the helmet–mask measuring system.

Secondly, the test subject stood on aluminum paper using the helmet-mask measuring system and wearing regular safety boots (Figure 3). The measurement method of helmet-mask measuring system was published earlier [4], and the method was approved by the local Ethical Committee (Pirkanmaa Health District, Finland, decision R05041). This method was used in order to see how much the helmet-mask system alters the conductivity of the test subject. This information is helpful in determining how well the system is able to measure the actual occupational exposure of a worker.

**Figure 4:**
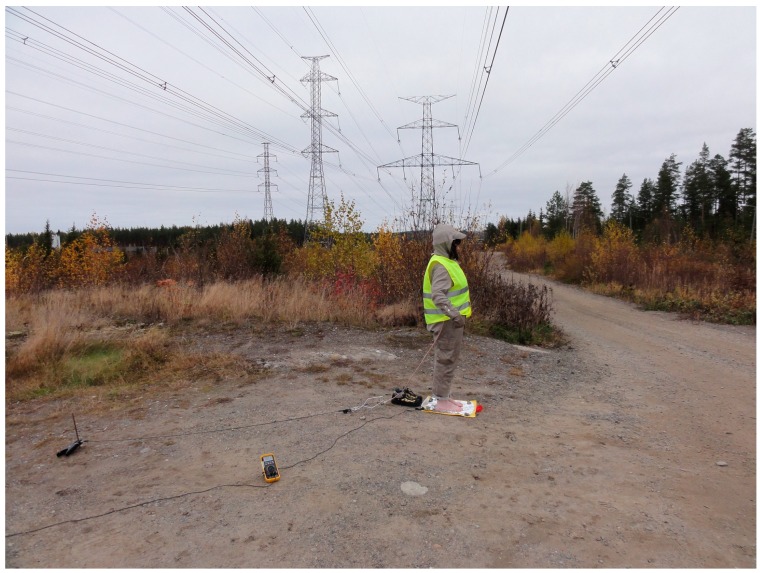
Test subject with grounded arc flash personal protective equipment (PPE).

**Figure 5:**
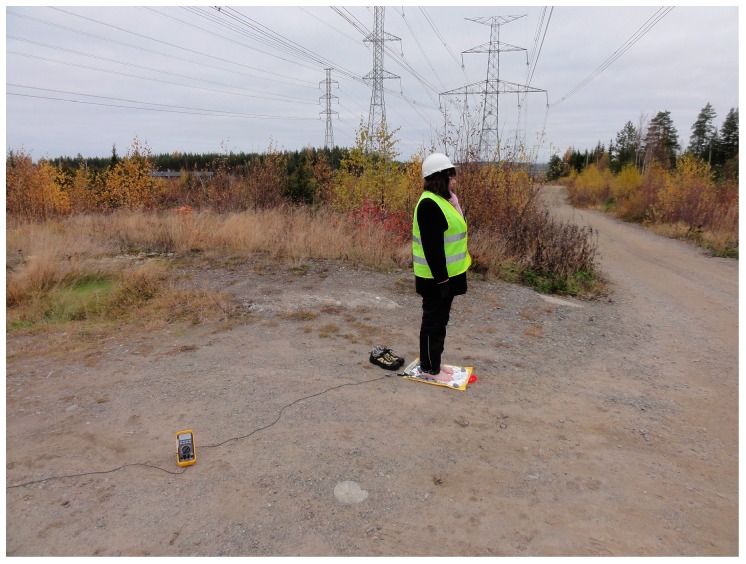
Measurements with normal clothes.

Thirdly, the test subject stood on aluminum paper, which was isolated from the ground with a plastic bag, while wearing different suits (Figure 3, Figure 4 and Figure 5). The current between the ground and the aluminum paper was measured with a Fluke 88-V/A multimeter (Fluke Corporation, Everett, WA, USA). Shoes were not used because the aim of the measurements was to provide an estimation of the current induced within a worker’s body. The measured current between the test subject and the ground can be used to estimate the induced current, and shoes would provide too much insulation for relevant estimations to be made.

The arc flash PPE was manufactured in Italy by Carraro S.R.L. [16], and it was rated to allow live work at nominal voltages of 800 kV AC and 600 kV DC. The PPE set included coveralls, a hood with a facial shroud, gloves, and socks. The PPE is made out of a fabric with polyamide fibers covered in a silver coating. The PPE, therefore, creates an effective Faraday cage around the wearer, which is meant to prevent a lethal electric current from going through the wearer´s body during an arc flash. Because of the formed Faraday cage, the PPE should also significantly reduce the electric field strength inside the PPE, thus shielding the wearer from occupational exposure as well. The patent associated with this PPE [16] does not describe the reduction of exposure to electric fields as its purpose. However, it cites an older patent, which does describe the reduction of exposure to electric fields as its purpose. Figure 5 shows the measurements with normal clothes.

## 3. Results

The measurements were performed on two separate days because the arc flash PPE was not available for use during the first scheduled measurement day. A total of 7 measurements were taken, one for each set of clothing. On the first measurement day, the electric field strength was 4.5 kV/m (height 1.7 m); on the second day, it was 4.6 kV/m (height 1.7 m). On both days, the air temperature was between 5 °C and 11 °C, and the relative air humidity ranged from 66% to 74%.

Table 1 shows the currents between the ground and the aluminum paper. The first result is a measurement to determine the baseline current for the aluminum paper when humans are not present. As mentioned, the current between the test subject and the ground can be used to estimate the induced current inside the body of test subjects. The current inside the test subject is of course larger than the currents measured because of the added resistance of the lower parts of the foot where the current must pass through to the aluminum paper. An induced current also flows on the surface of the test subject, which does not contribute to the internal current but can be seen in the measurement results.

When the test subject used the helmet-mask measuring system, the current measured by the system was 52.2 μA, and the current between the ground and the aluminum paper was 50.0 μA. When the test subject wore a regular helmet, normal clothes, and no socks, the current between the ground and the aluminum paper was 64 μA. When she used the PPE (with grounding), the current between the ground and the aluminum paper was 6.1 μA. This was a 90.5% reduction in the measured current.

## 4. Discussion

In the comparison between the measurement method (use of aluminum paper) and the helmet-mask measuring system, the currents were quite similar (the current between the ground and the aluminum paper was 50.0 μA, and the total contact current of the helmet-mask measuring system was 52.2 μA). 

In an earlier study [5], the measured current between the ground and the aluminum paper was 41.4 µA when the test subject wore normal clothes without shoes or socks. In this study, the current was 64 µA with very similar clothing. This can be partly attributed to the larger electric field strength present during the new measurement, which was between 4.6 and 4.7 kV/m, compared to the electric field strength of 3.8 kV/m in the other study (an 18% difference). Higher relative air humidity might also have played a role. Another explanation could be the possible difference in the successful grounding of the aluminum paper at the beginning of the measurement.

The use of arc flash PPE was very successful in reducing the induced current between the aluminum paper and the ground. The induced current was reduced to 10% of the value measured while wearing normal clothes. In the earlier experiment [5], the protective coveralls came from Japan. The full suit included a hat, a jacket, trousers, gloves, and socks, which were electrically connected to each other and grounded at the soles. They were made of a conductive textile, but they were not arc flash PPE, i.e., they were only meant to decrease the electric field exposure. The PPE tested in this experiment was arc flash rated PPE, making them suitable for electricians who work in high voltage areas.

The contact currents measured were significantly lower than the ICNIRP reference level of 1 mA, which is due to the relatively low strength of the electric field under the power line. Significantly higher electric field strengths can be found in electrical substations, where the contact currents would be correspondingly higher than in this study. More measurements at electrical substations should be taken in the future.

The usefulness of the PPE is hindered by the fact that it has to be grounded by a conductor wire. This limits worker mobility and range to the length of the conductor. To limit the exposure to a minimum, the worker should also use the shrouded hood of the PPE, which limits visibility and restricts breathing. The PPE can also significantly increase the heat load of the worker during hot days.

The earlier study [5] found that most clothes have very little effect on the measured induced current through the body, which was replicated in this study. Similar results were observed in an experiment conducted in 2015 [6], where most of the tested jackets had very little effect on the measured electric field strength. It would seem that, in order to significantly reduce the exposure to large induced currents through the body, a specially designed conductive suit that is grounded is required.

The achieved results may not take into account either short-term measurement variability or variability in electric field strength at different times of the year. The measurements in the present paper should be seen as a pilot study; more time points and repeated measurements per condition should be added to future studies so that the experiments can be conducted under more realistic (higher) exposure conditions experienced by electricians.

## 5. Conclusions

Based on our measurements, we can conclude that the arc flash PPE is one possible way to effectively decrease workers’ exposure to electric fields. Since the highest measured contact currents were significantly lower than the ICNIRP reference level of 1 mA, workers should be safe from occupational exposure at ground level under 400-kV power lines even without a PPE. However, the PPE can be somewhat difficult to use because workers need to ground the coveralls and preferably use the facial shroud, which restricts breathing. It can also be useful in workplaces where spark discharges can be of nuisance, as the PPE can conduct the discharge currents to the ground without the test subject even noticing them.

## Figures and Tables

**Table 1:** Measured electric field strengths (kV/m) and current between the ground and the aluminum paper (μA) with a helmet-mask measuring system or different clothes.

E_rms_^1^ (kV/m)	Current between Ground and Aluminum Paper (µA)	Clothing Worn
4.5	2.7	Aluminum paper baseline
4.5	50.0	Helmet-mask measuring system, regular safety boots
4.5	59.0	Normal clothes, woolly socks
4.5	62.0	Normal clothes, no socks
4.5	64.0	Normal clothes and a helmet, no socks
4.6	6.1	Arc flash PPE, grounded
4.6	69.2	Arc flash PPE, not grounded

^1^ E_rms_: The local electric field strength during the measurement.
